# Six-month outcomes of the HOPE smartphone application designed to support treatment with medications for opioid use disorder and piloted during an early statewide COVID-19 lockdown

**DOI:** 10.1186/s13722-022-00296-4

**Published:** 2022-03-07

**Authors:** Jacqueline Hodges, Marika Waselewski, William Harrington, Taylor Franklin, Kelly Schorling, Jacqueline Huynh, Alexa Tabackman, Kori Otero, Karen Ingersoll, Nassima Ait-Daoud Tiouririne, Tabor Flickinger, Rebecca Dillingham

**Affiliations:** 1 Division of Infectious Diseases and International Health, University of Virginia, PO Box 801340, Charlottesville, VA 22908-1340 USA; 2 University of Michigan Medical School, 7300 Medical Science Building I - A Wing, 1301 Catherine St., Ann Arbor, MI 48109-5624 USA; 3 University of Virginia School of Medicine, 1215 Lee St, Charlottesville, VA 22903 USA; 4 Department of Psychiatry and Neurobehavioral Sciences, University of Virginia, 1300 Jefferson Park Ave., Charlottesville, VA 22903 USA; 5 Department of Medicine, University of Virginia, 1215 Lee St, Charlottesville, VA 22903 USA

**Keywords:** Opioid use disorder, Medications for opioid use disorder, Mobile health, Digital health

## Abstract

**Background:**

Morbidity and mortality related to opioid use disorder (OUD) in the U.S. is at an all-time high. Innovative approaches are needed to address gaps in retention in treatment with medications for opioid use disorder (MOUD). Mobile health (mHealth) approaches have shown improvement in engagement in care and associated clinical outcomes for a variety of chronic diseases, but mHealth tools designed specifically to support patients treated with MOUD are limited.

**Methods:**

Following user-centered development and testing phases, a multi-feature smartphone application called HOPE (Heal. Overcome. Persist. Endure) was piloted in a small cohort of patients receiving MOUD and at high risk of disengagement in care at an office-based opioid treatment (OBOT) clinic in Central Virginia. Outcomes were tracked over a six-month period following patient enrollment. They included retention in care at the OBOT clinic, usage of various features of the application, and self-rated measures of mental health, substance use, treatment and recovery.

**Results:**

Of the 25 participants in the HOPE pilot study, a majority were retained in care at 6 months (56%). Uptake of bi-directional features including messaging with providers and daily check-ins of mood, stress and medication adherence peaked at one month, and usage persisted through the sixth month. Patients who reported that distance to clinic was a problem at baseline had higher loss to follow up compared to those without distance as a reported barrier (67% vs 23%, p = 0.03). Patients lost to in-person clinic follow up continued to engage with one or more app features, indicating that mHealth approaches may bridge barriers to clinic visit attendance. Participants surveyed at baseline and 6 months (N = 16) scored higher on scales related to overall self-control and self-efficacy related to drug abstinence.

**Conclusions:**

A pilot study of a novel multi-feature smartphone application to support OUD treatment showed acceptable retention in care and patient usage at 6 months. Further study within a larger population is needed to characterize ‘real world’ uptake and association with outcomes related to retention in care, relapse prevention, and opioid-associated mortality.

**Supplementary Information:**

The online version contains supplementary material available at 10.1186/s13722-022-00296-4.

## Background

Opioid use disorder (OUD) remains a significant public health crisis in the United States, where over 2 million people are living with OUD [1]. Fatal overdose rates have been climbing since 2019 and have reached an all-time high in the midst of the COVID-19 pandemic [2, 3] driven primarily by synthetic opioids, including illicitly manufactured fentanyl. These concerning trends are mirrored within the state of Virginia, where fatal overdose remains the leading cause of unnatural death since 2013, and where overdose deaths have increased by over 40% between 2019 and 2020 [4].

Treatment with medications for opioid use disorder (MOUD) is an evidence-based approach, combining medications with counseling and behavioral therapy. Retention in treatment with MOUD is associated with decreased drug use, improved quality of life and social functioning, overdose prevention and reduced mortality [5–9]. Despite the efficacy and increased accessibility of buprenorphine/naloxone through officebased opioid treatment (OBOT) programs, patient retention in care remains highly variable across programs [5]. Previous studies indicate that a retention rate of 50% or higher at 12 months is a marker for a successful treatment program in most high-income countries, whereas acceptable shorter-term retention is less defined, particularly for patients facing different barriers to care [6, 10].

Mobile health (mHealth) strategies have demonstrated benefit for improving patient engagement and longitudinal retention in care for a variety of chronic illnesses [11–16]. Several mHealth interventions designed to support recovery have demonstrated usability within substance-using populations [17] as well as association with improvement in outcomes, including abstinence and relapse prevention [18–20] and outpatient treatment utilization [21] for patients with alcohol use disorders. Two studies extending use of a multi-feature smartphone app to support engagement in treatment of several different substance use disorders showed improved retention in mandated [22] and post-residential [23] outpatient treatment.

Mobile platforms designed specifically to support MOUD have included features like medication adherence monitoring and supervision [24], self-monitoring including substance use, cravings, and triggers [25], educational resources [26] and delivery of cognitive-behavioral therapy to supplement outpatient treatment [27]. Various platforms designed to support MOUD are currently being tested [28], however, evidence of impact of mHealth interventions to support MOUD on sustained retention in treatment remains limited [29].

We designed and pilot tested a multi-feature mHealth intervention tailored specifically for deployment in association with MOUD at an OBOT clinic in Central Virginia at high risk of disengagement in services. The HOPE smartphone app (Heal. Overcome. Persist. Endure.) demonstrated high usability and acceptability following user testing within our pilot study cohort [30]. In this report, we examine the primary outcome of retention in care at 6 months by pilot study participants, and characterize participant app usage over that time period. We also report on patient-rated psychosocial measures of mental health, substance use, treatment and recovery.

## Methods

### Study setting

The University of Virginia OBOT Clinic opened at the end of 2015. The clinic serves a primarily nonurban adult patient population, providing MOUD including prescription medication management (buprenorphine/ naloxone and naltrexone) along with counseling, case management, overdose education and other services. Patients are referred from counties across Central Virginia, with patient drive time as far as 2 h each way from the clinic. Transportation support services are offered through Medicaid for covered patients. Eligibility for treatment with MOUD at the clinic includes age of 18 years or older, with a diagnosis of moderate or severe opioid use disorder, or any severity of opioid use disorder if pregnant. The study received approval from the University of Virginia Institutional Review Board.

### HOPE platform development

**Fig. 1:**
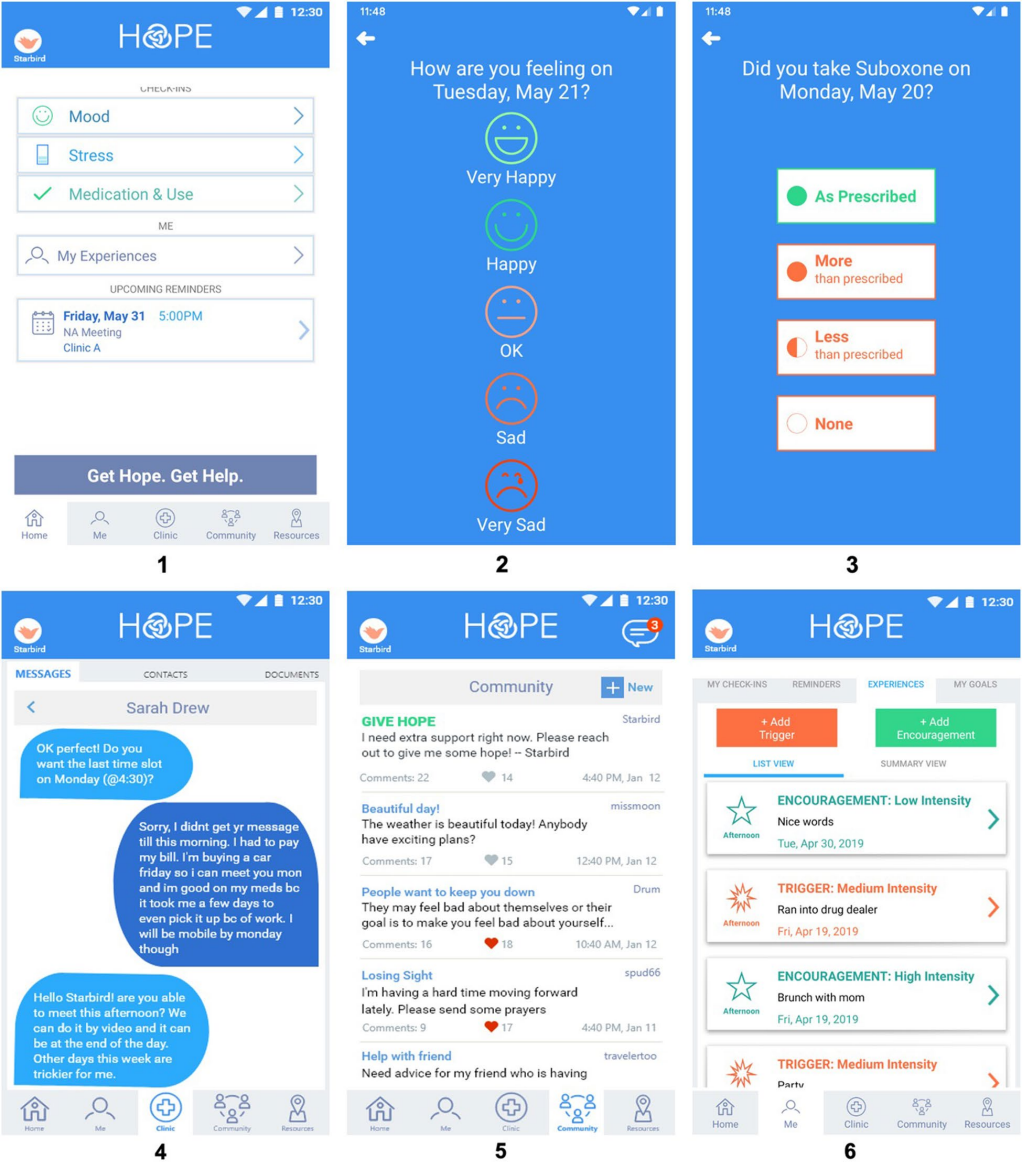
Screenshots of select HOPE features (demo accounts). Features include a dashboard (**1**) daily check-ins of mood (**2**), stress, medication (buprenorphine/naloxone) (**3**) and substance use, provider messaging (**4**), a community board for anonymous peer messaging (**5**), goals, and experiences encountered including triggers and encouragements (**6**)

The formative development of the HOPE participant app and provider web platform is described in detail in a separate publication [30]. In short, participant and provider interviews were conducted during the formative phase to elicit participants’ current self-monitoring practices and experiences as well as barriers and needs surrounding OUD treatment and recovery. The platform includes features selected from a previously built, evidence-based mobile health strategy to support chronic care [31] as well as development of new features to support substance use recovery specifically. Additional feature selection was guided by participant interviews. The platform was named HOPE: Heal. Overcome. Persist. Endure. based on participant feedback. Final features were iteratively refined using rounds of participant interviews where participants reviewed wireframe and high-fidelity mockups. The final list of platform features is summarized (see Additional file 1), with selected screenshots of the HOPE interface shown in Fig. 1.

### Study enrollment

Pilot study enrollment occurred between October and December 2019. Participants and providers were enrolled from the University of Virginia OBOT Clinic. All clinic providers were eligible for participation, and if they accepted enrollment, they were trained by study staff on study procedures and provided informed consent. Three providers (1 physician, 1 nurse and 1 social worker) underwent training on use of the app interface. Throughout the study, providers could access a web-based portal for review of participant responses or for participant messaging. No formal incentives were given to providers for participation. Patient participants were offered enrollment during routine outpatient visits to the OBOT clinic. Inclusion criteria included patients eligible for MOUD who were at least 18 years old. Participants were additionally screened prior to enrollment by the clinic’s social worker for one or more risk factors for disengagement in care including: failure to meet one or more basic needs (homelessness, lack of access to transportation, lack of phone access), high risk substance use history (endorsing ongoing active substance use, history of multiple overdoses, social connections with active substance users, transitioning from residential to outpatient treatment), and active mental health concerns determined by social worker assessment. Participants without smartphone access at enrollment were provided a smartphone as well as a prepaid account with a service provider. Participants were trained on app functionalities by study staff at enrollment. All participants were enrolled for at least six months during which they were free to use the mobile platform as they wished. Participants were allowed to continue using the app after their six-month follow-up, but all accounts were deactivated following the end of the study (July 2020). Throughout the pilot, study staff regularly monitored platform content, responded to participant questions on the messaging feature, and provided occasional prompts for discussion in the community board.

### Data collection

#### Baseline characteristics

Participant baseline characteristics including sociodemographic information, distance from residence to the OBOT clinic at enrollment, and substance use history were collected using electronic surveys upon pilot study enrollment. Participants were also surveyed on perceived barriers to clinic visit attendance, including distance to the clinic (To what extent is the distance from your home to this clinic a problem for you?), as well as access to transportation (To what extent is access to personal or public transportation from your home to this clinic a problem for you?). Response options included: No problem at all, slight problem, somewhat of a problem, and significant problem. Housing status was also assessed (Which of the following best describes your current living situation?). Participants were categorized as housing stable if they selected ‘Own an apartment or house’ or ‘Rent a room, apartment or house,’ and housing unstable if selected ‘Temporarily staying with others,’ ‘Temporarily in transitional housing program,’ ‘In shelter for homeless people,’ or ‘On the street or outside.’ Participant days in care at the clinic prior to study enrollment were obtained by chart review.

#### HOPE app usage

Participants’ app usage data were evaluated over the six months following their enrollment in the study. Individual-level data were automatically collected for each user through the app and downloaded for analysis. Features examined included direct provider messaging, the community board, daily queries or ‘check-ins,’ experiences, and goals.

#### Treatment outcomes

The primary outcome of retention in care at the OBOT clinic during the six-month post-enrollment period was obtained by chart review. Retention in care was defined as a participant’s documented attendance to at least one visit in the clinic during the 6-month period following the participant’s enrollment date, as well as a subsequent visit following the 6-month post-enrollment date at the time of chart review (December 2020). Loss to follow up was defined as either dismissal from the OBOT clinic due to violation of clinic policies, or a cessation of appointment attendance during the six months following enrollment in the study with no return to clinic prior to the time of chart review in December 2020. Data for participants’ urine drug screens (UDS) were also abstracted from the electronic medical record.

Electronic surveys were distributed to participants on the date of study enrollment (baseline) and upon six-month follow up visits to assess additional outcomes of interest. Self-scored surveys included scales related to self-control and self-efficacy surrounding substance use, specifically the Brief Self Control Scale (B-SCS) [32] and the Drug Abstinence Self-efficacy Scale (DASE) [33]. Survey items assessing mental health and stress were also included: the five-item Mental Health Inventory (MHI-5) [34] and the Perceived Stress Scale (PSS) [35]. Additional surveys assessed participant perceptions of support, provider empathy, and stigma using the Medical Outcomes Study Social Support Survey Instrument (MOS-SSS) [36], the Consultation and Relational Empathy Measure (CARE) [37], and the Perceived Stigma of Substance Abuse Scale (PSAS) [38], respectively.

#### Statistical analyses

Descriptive statistics were used to analyze baseline characteristics, app usage by feature, and participant treatment outcomes. Mean total feature usage was compared between the groups of participants lost to follow up by month six and those retained in care for each bi-directional feature (direct provider messaging, community board, daily check-ins) using independent t tests. Mean total feature usage per user during the six-month period was compared across subgroups by baseline characteristics including gender, housing stability status, distance score, transportation score (independent t tests), and race/ethnicity (one-way anova). Rates of loss to follow up by six months were compared across subgroups by baseline characteristics including gender, race/ethnicity, housing stability and distance and transportation scores (Chi square tests). Mean distance in miles from the participant’s documented place of residence to the OBOT clinic was compared between participants who were lost to follow up and those retained in care (independent t test). Participant scores for the selected surveys were compared across the baseline (upon enrollment) and 6-month timepoints (paired t-tests). Pearson correlation was used to examine the association of mean total usage of bi-directional features during the 6-month period with changes in participant self-scoring for multiple scales from baseline to six months, including the frequency of app logins during the period the server was active (October 2019 to July 2020). Analysis was performed using IBM SPSS Statistics for Mac, Version 26.0 (Statistical Package for the Social Sciences, IBM Corp, Armonk, NY, USA).

## Results

### Participant baseline characteristics

Baseline characteristics for the 25 participants enrolled in the pilot study are summarized (Table 1). All patients who were offered enrollment accepted. On average, participants were approximately 34 years old and 52% were male. The majority (84%) identified as White/Non-Hispanic, while the remainder were categorized as ‘Other’ due to small group size and concern for participant privacy (identified as Black/Non-Hispanic, American/Indian/Alaska Native, and Multiple). Eight participants owned a smartphone prior to enrollment. Participants were enrolled a median of 75 days after establishing care at the clinic. Upon enrollment, participants lived approximately 24 miles from the clinic on average. Over half of the cohort reported housing instability of some kind.

**Table 1:** Baseline characteristics for participants (N = 25)

	N (%) or Mean (SD)
Age	34 (8)
Gender, male	13 (52%)
Race/Ethnicity	
White, non-Hispanic	21 (84%)
Other	4 (16%)
Housing	
Stable housing	11 (44%)
Unstable housing	14 (56%)
Education	
Less than high school	5 (20%)
High school or GED	12 (48%)
Any college	8 (32%)
Employment	
Employed full or part-time	10 (40%)
Receiving disability benefits	4 (16%)
Unemployed	11 (44%)
Owned a smartphone prior to study enrollment	8 (32%)
Distance from OBOT clinic (miles)	24 (21)
Distance to clinic self-rated as:	
No problem at all	13 (52%)
Slight problem	9 (36%)
Somewhat of a problem	1 (4%)
Significant problem	2 (8%)
Transportation self-rated as:	
No problem at all	14 (56%)
Slight problem	4 (16%)
Somewhat of a problem	1 (4%)
Significant problem	6 (24%)
Time in OBOT clinic (days, Median [IQR])	75 [42–134]

Demographics obtained during baseline assessment upon enrollment in the study. Time in OBOT clinic describes the number of days between a participant establishing care and the date of enrollment in the study

### Participant app usage

**Fig. 2:**
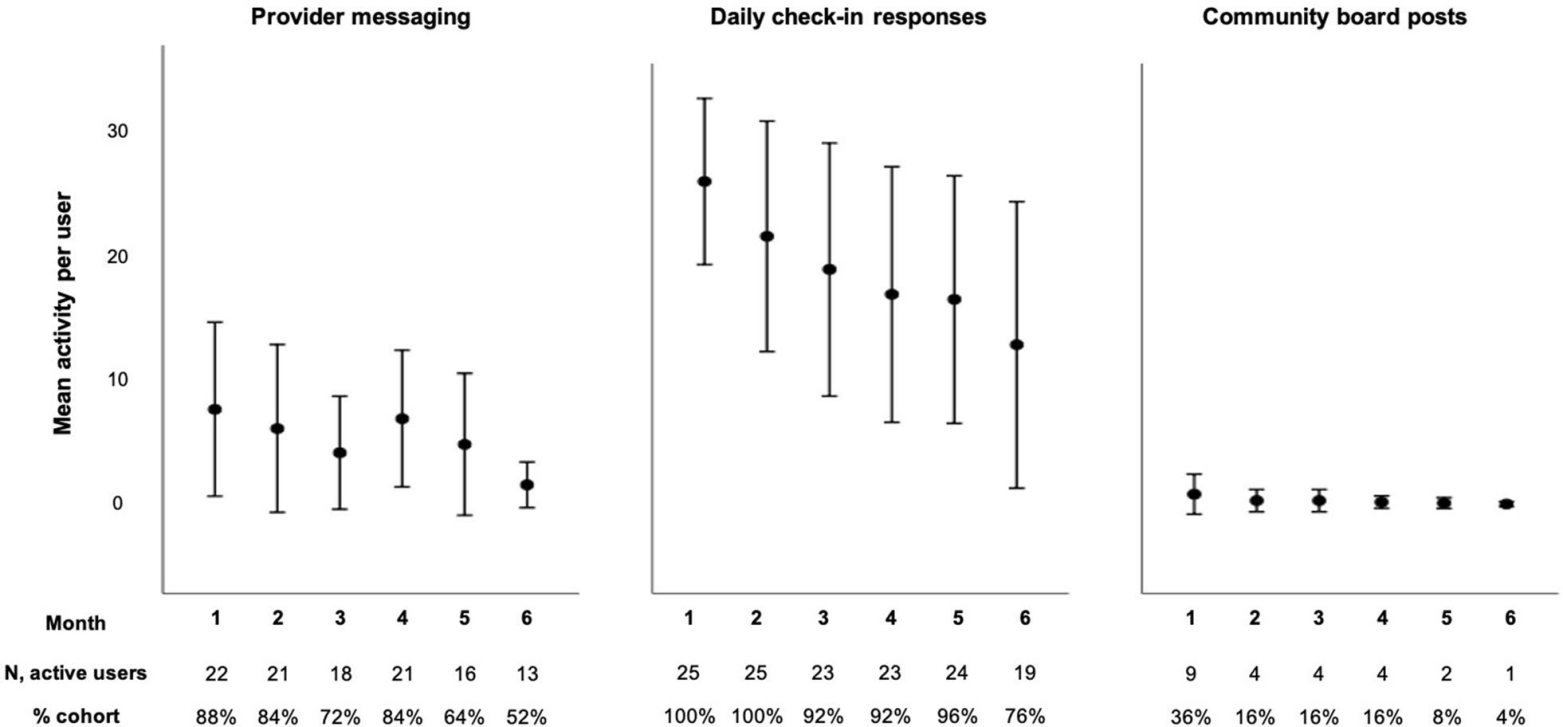
Cohort HOPE app activity. Participant activity is averaged by feature for cohort participants for each month following enrollment. Active user defined as participant using respective feature one or more times in a given month

Participant usage of selected HOPE app features is graphed in Fig. 2 (total user activity per month is summed for each participant and averaged across the cohort) and is summarized in Additional file 2. The majority of participants used the provider messaging and daily check-in features at least once during the first month of the study (88% and 100%, respectively). Over half of participants continued to use the provider messaging feature by the sixth month, whereas more than three quarters (76%) continued to use the daily check-in feature during the sixth month.

Initial cohort uptake was lower for the community board and goals features (36% with active use of both features during month 1) and moderate for the experiences feature (52% of the cohort with active use during month 1). Following the first month, cohort usage of all three features dipped significantly, with little to no active use of the goals and experiences features by month six (active use ranged from 0–8% of the cohort for months 2–6).

**Fig. 3:**
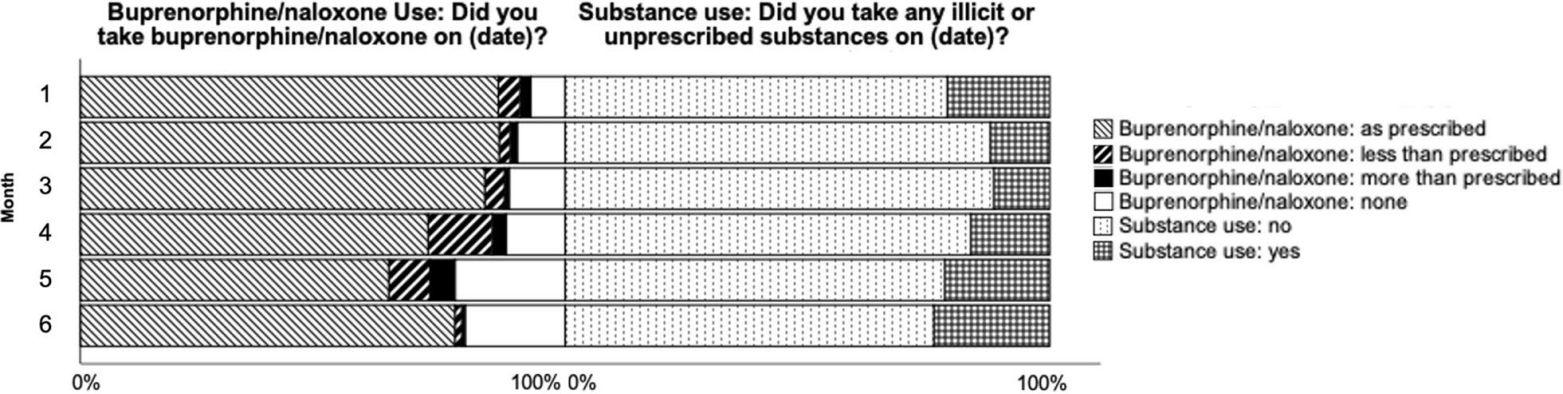
HOPE daily check-ins for buprenorphine/naloxone use and substance use. Mean proportion of each response type to daily check-ins sent by active users are listed for each month following their enrollment

Participant responses to daily check-ins related to buprenorphine/naloxone use and substance use were also examined by response type (Fig. 3). Responses were not visible to providers or study staff during the study period, and served primarily as a self-monitoring tool. For the majority of the responses sent for buprenorphine/naloxone use each month, participants endorsed taking buprenorphine/naloxone ‘as prescribed’ throughout the six months. The proportion of substance use check-ins (querying any use of illicit or unprescribed substances) sent by participants with affirmative responses (responding ‘yes’) each month ranged from 12 to 24% and was overall fairly stable between the first and sixth months, with between 5 to 11 participants responding affirmatively per month over the six-month period.

### Retention in care

At 6 months post-enrollment, 56% of the cohort was retained in care. Of the 11 participants who established care within 2 months of enrollment in the study, 7 (64%) were retained in care. Loss to follow up at the OBOT clinic occurred for 11 of the 25 participants (44%) by 6 months post-enrollment. Three participants were dismissed due to contract violations, and 8 stopped attending visits prior to expected follow up at six months. Eight participants (32%) tested positive one or more times for opioids on UDS performed in the six months following enrollment. The average time to first positive UDS post-enrollment was 1.98 months (SD 1.19).

When comparing app feature usage for participants lost to follow up (N = 11) and those retained in care (N = 14), mean total feature usage during the six-month period was lower on average for all three bi-directional features for those lost to follow up, though the differences were not statistically significant (lost to follow up versus retained group: 24 versus 38 direct messages, p = 0.1; 0.9 vs 2.6 community board posts, p = 0.3; 95 vs 121 check-in responses, p = 0.2). All 11 participants lost to follow up used at least one app feature following their last documented encounter at the clinic. Nine of the 11 participants used at least one feature following their last encounter in each month up to the end of the six-month post-enrollment period examined. All of those 9 participants used the check-in feature every month, and 4 also continued to use the messaging feature at least once every month after loss to clinic follow up. Only one participant used the community board after loss to clinic follow up, less than two weeks following enrollment, and used it once each month for two months.

### Factors associated with retention in care and app usage

Rates of retention in care at the OBOT clinic were analyzed by baseline characteristics. There was no statistically significant difference in the proportion lost to follow up when comparing subgroups by gender or race/ethnicity. Participants who rated distance from the clinic as ‘no problem’ when surveyed were, however, significantly less likely to be lost to follow up (3/13 participants or 23%) compared to those indicating any problem with distance (‘slight problem, somewhat of a problem, significant problem’, 8/12 or 67%, p = 0.03). Distance in miles from residence to clinic was lower on average for participants retained in care versus those lost to follow up, though not significant (18.9 versus 30.6 miles, p = 0.2). Participants who rated access to transportation as ‘no problem’ were less likely to be lost to follow up (4/14 or 29%) compared to those indicating any problem with transportation (7/11 or 64%, p = 0.08), as were participants who endorsed stable housing at baseline (3/11 or 36%) compared to those with unstable housing (7/14 or 50%, p = 0.5), though these differences were also not significant. For bi-directional features (provider messaging, community board, and daily check-ins), mean total usage per participant during the six-month period did not differ significantly for any feature examined between subgroups stratified by any of the aforementioned baseline demographics.

### Additional treatment outcomes

**Fig. 4:**
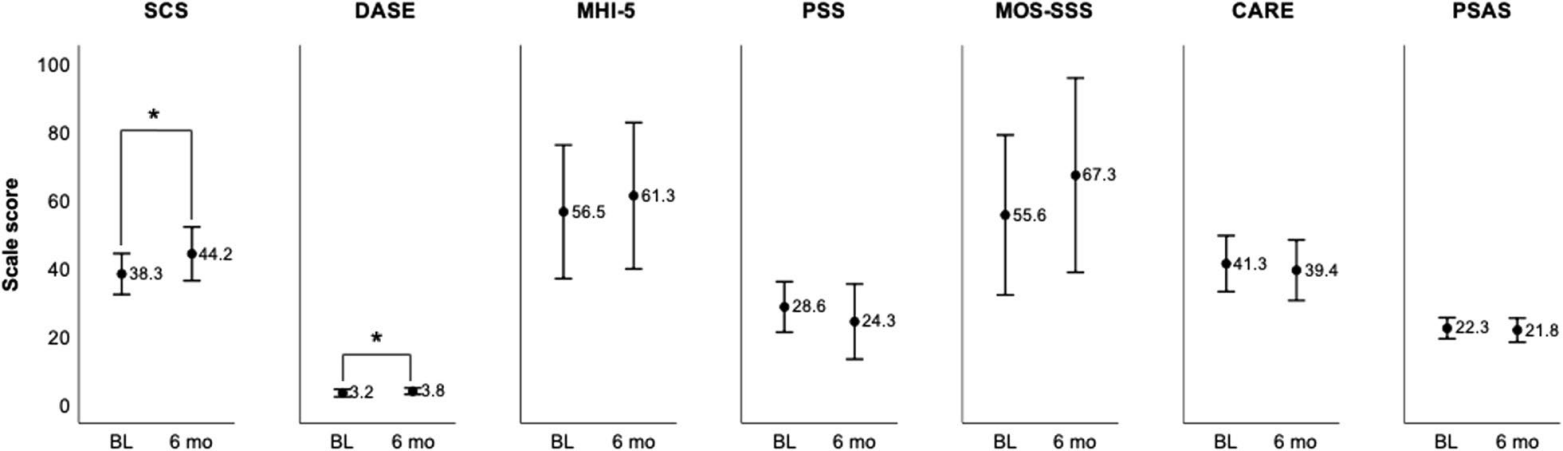
Scale scoring comparisons, baseline and six months. Scales were all self-scored by participants and averaged for those with available data at both timepoints (N = 16 for all surveys except CARE, N = 14). *SCS* Self Control Scale (score range: 13–65), *DASE* Drug Abstinence Self-efficacy Scale (1–5), *MHI-5* Mental Health Inventory (0–100), *PSS* Perceived Stress Scale (0–40), *MOS-SSS* Medical Outcomes Study Social Support Survey Instrument (0–100), *CARE* Consultation and Relational Empathy Measure (10–50), *PSAS* Perceived Stigma of Substance Abuse Scale (8–32). *p = 0.02

Participant self-scoring for selected electronic surveys was compared for participants with data at both the baseline and 6-month timepoints (Fig. 4). Compared to baseline, scores were higher on average at month six for the Brief Self Control and Drug Abstinence Self-Efficacy Scales, the Mental Health Inventory, and the Medical Outcomes Study Social Support Survey. The Brief Self Control and Drug Abstinence Self-Efficacy scales demonstrated statistically significant increases in mean scores between the baseline and six-month assessments (p = 0.02). Mean scores declined (i.e. improved) for the Perceived Stress Scale and Perceived Stigma of Substance Abuse Scale, but also declined for the Consultation and Relational Empathy Measure, however none of these differences were statistically significant.

The association between mean total usage of features per user and changes in scores for participants with survey data at both timepoints (N = 16) was examined for the Brief Self Control Scale (B-SCS) and the Drug Abstinence Self-Efficacy Scale (DASE). The frequency of participant app logins while the server was active demonstrated a moderate positive correlation with changes in users’ average B-SCS score from baseline to six months (r = 0.50, p = 0.049). When mean total usage of the bi-directional features per user was examined only for the six-month periods following participants’ enrollment, usage weakly positively correlated with changes in the B-SCS scale (direct messages, r = 0.3, p = 0.3; community board posts, r = 0.05, p = 0.9) or was moderately positively correlated but not statistically significant (check-in responses, r = 0.5, p = 0.05). Usage was not significantly correlated with changes in the DASE scale between those timepoints for any of the bi-directional features during six-month post-enrollment periods, or for app log-ins performed during the total duration the server was active.

## Discussion

This study demonstrated early uptake and associated retention in care for the majority of a cohort that had the opportunity to use a multi-feature clinic-associated smartphone app designed specifically to support treatment with MOUD. Multiple functionalities related to treatment with MOUD were piloted in this study, including direct and secure provider in-app messaging and peer messaging on a community board, and uptake varied highly by app feature.

Over the six months following enrollment, cohort participants demonstrated the highest and most consistent uptake of the provider messaging and daily check-in features. The majority of participants demonstrated ongoing entry of responses to both substance use and buprenorphine/naloxone use check-ins by month six. These check-ins were designed around patient feedback obtained during the formative phase [30], and usage suggests participants found it usable for the purpose of tracking their substance use or conversely, abstinence from substances, as well as use of their buprenorphine/naloxone. Additionally, check-ins appear automatically upon logging into the app, making this feature very low barrier for patient uptake**.** Usage of the remaining features we examined was lower, including for the community board, experiences, and goals features. Notably, participants had expressed favorable perceptions of the community board during early app testing. At one month, they wished the board had higher activity [30]. Given this pilot study was conducted in a small cohort with enrollment staggered over several months, it is likely that inadequate collective activity on the board discouraged use of the feature over the follow-up period, despite participants finding the feature highly desirable during the formative development phase. Based on our review of app usage surrounding the date of the first COVID-19 ‘stay at home’ executive order (51) in Virginia (March 12, 2020), there was a small increase in messaging activity per user on average in the month following that date when compared to activity the month prior to that date (5.9 ± 6.4 vs 4.1 ± 5.0, respectively). Community message board activity was too low overall to make meaningful observations by month.

For the primary outcome of retention in care, a majority of study participants were retained by six months, half of whom (7/14) were enrolled early in the course of establishing care with the OBOT clinic, a critical point in recovery when patients are often at highest risk of relapse and/or disengaging in care [39, 40]. Participants for this cohort were identified as high risk for early disengagement, and previously identified thresholds for acceptable retention in treatment (e.g. 50% at 12 months) [6, 10] may be less applicable. Participants retained in care were on average more active on the app, and while the difference was not significant when compared to those lost to follow up, it suggests that engaging with the app may support participants’ engagement with their overall treatment. It is possible that high early app engagement may support a more robust initial response to treatment programs in a larger population. Multiple features may contribute to delivery of a higher intensity, frequency and diversity of services, a strategy which has been shown to promote treatment retention and positive post-treatment outcomes [41].

The soaring rates of opioid overdose during the COVID-19 pandemic and associated service disruptions have encouraged broader discourse on increasing access to MOUD for patients in remote areas or who are unable to attend clinic visits regularly despite continuing desire to engage in care [2]. While the OBOT clinic began to offer telemedicine visits to patients on a case-by-case basis during the study period due to COVID-19 lockdowns (April to July 2020), the majority of clinic visits monitored for this study by chart review were conducted in-person (only one participant’s chart documented a telemedicine visit during the study period). Retention in care was notably impacted by participants’ perceptions of their distance required to travel to the clinic when surveyed at baseline, demonstrating that while a mHealth app like HOPE may improve participants’ ability to stay engaged in their recovery process through app-based functionalities, ultimately patients who must travel too far to keep up with the requirements of treatment (e.g. in-person follow-up with UDS testing) will likely struggle with remaining on MOUD. Continued activity on the check-in and provider messaging features following participants’ last in-person visits for those lost to follow up suggests that participants continued to engage in self-monitoring and communicate with clinic providers despite barriers to in-person attendance at clinic visits. These findings suggest that tools like HOPE could be used to maintain therapeutic relationships and even re-engage participants who might relapse or transiently disengage in care for different reasons, despite an ongoing desire to continue treatment and achieve recovery. Functionalities like low barrier communication with clinic providers and support staff provided by mHealth tools may be helpful for identifying structural barriers as they arise in real time. However, mHealth strategies may provide the most benefit in combination with newer, more flexible models of care. Specifically, they will likely be insufficient to retain individuals in care without coincident attention to overcoming structural barriers to care, such as housing instability and poor transportation access.

Finally, when surveyed on a variety of psychosocial and substance use measures, a subset of participants with responses at enrollment and 6 months showed non-significant improvement in scores between those timepoints for all measures except the CARE measure relating to perceptions of provider empathy, which also had fewer surveys available at both timepoints. Notably, both the Brief Self Control Scale (B-SCS) and Drug Abstinence Self-Efficacy Scale (DASE) showed significant improvement for this subset of participants, and B-SCS score improvement correlated moderately with the frequency of participant app log-ins. It is important to note that these trends in scale scoring were detected while testing multiple comparisons, for a small subset of participants that had scores at both timepoints, only two of whom were lost to in-person clinic follow up, which may bias results toward favorable score changes. Still, the trends suggest that the subset that did respond at both timepoints and that remained engaged in treatment may have subjectively experienced enhanced overall self-control, as well as increased self-efficacy specifically related to abstinence over the course of the study.

Several limitations exist for this study. This single-arm study was conducted for a small cohort, with limited power to detect differences in outcomes of interest between subgroups while adjusting for covariates including participants’ active app usage and various demographics like race/ethnicity, gender, and various pre-identified risk factors for disengagement. The lack of standardized definitions for retention in treatment with MOUD, particularly during periods of COVID-19 related lockdowns when there was some increased flexibility in treatment requirements, limited our ability to track retention using criteria validated for this population. Relapse prevention is an additional important outcome when studying patient participation in treatment with MOUD, and while post-enrollment UDS data was available for participants upon chart review, urine testing frequency was highly variable in the pre- and post-enrollment periods because participants were recruited at varying points in their treatment course and urine testing requirements varied accordingly. Direct comparisons in rates of screen positivity for substances thus could not be made consistently between those periods across participants. Additionally, while smartphones were provided to participants who needed them, smartphone access is not well documented for this patient population, and usage may not be widely generalizable to this clinic population or similar rural populations absent the provision of smartphones.

## Conclusions

In conclusion, the HOPE application was piloted in a small cohort at risk for disengagement in care, and showed acceptable rates of retention and application usage by six months, with highest usage demonstrated for features that support self-monitoring and out-of-clinic patient-provider communication. Patient-rated self-efficacy and self-control measures improved over the six-month period. Perceived distance to clinic as a barrier predicted in-person clinic attendance even for participants with access to the application. Nonetheless, given ongoing usage of the application following cessation of in-person attendance, it is possible that the HOPE app may offer an opportunity to provide and/or support more flexible treatment models aiming to expand access to MOUD. Further study in a larger cohort of patients enrolled in treatment with MOUD is needed to characterize the impact of ‘real-world’ smartphone accessibility on app uptake, as well as to study associations of app usage with outcomes related to retention in care, relapse prevention, and opioid-associated mortality.

## Supplementary Information

**Additional file 1**. List of HOPE platform features and functions. Table describing features of HOPE platform.

**Additional file 2**. HOPE app activity. Summary of participant activity on platform during 6-month post-enrollment period.

## Data Availability

The datasets generated during and/or analyzed for this study are available from the corresponding author on reasonable request.
