# Supplementary material for: Six-month outcomes of the HOPE smartphone application designed to support treatment with medications for opioid use disorder and piloted during an early statewide COVID-19 lockdown
Source: Addict Sci Clin Pract. 2022 Mar 7;17:16. doi: 10.1186/s13722-022-00296-4 (PMC8899792; doi:10.1186/s13722-022-00296-4)
Supplement: Supplementary file 1 — Additional file 1. List of HOPE platform features and functions. Table describing features of HOPE platform. [file 13722_2022_296_MOESM1_ESM.docx]

**Additional file 1:**

**List of HOPE platform features and functions.**

| **Feature** | **Description** |
| --- | --- |
| Check-ins | Daily queries asking participants about their mood, stress, buprenorphine/ naloxone adherence, and non-prescribed substance use |
| Get Hope. Get Help. | Emergency support system with access to uplifting quotes, request for support from the community, clinic contact number, crisis hotline, and 911 emergency number |
| My Check-Ins | Self-monitoring tool with check-in responses displayed over time |
| My Experiences | Allows participants to enter triggering and encouraging experiences |
| My Reminders | Sends a reminder notification for entered reminders or appointments |
| My Goals | Allows participants to enter and track progress towards recovery goals |
| Messages | Private, secure messaging between participants and providers or study team members |
| Documents | Allows participants to securely upload documents to share information with clinic providers |
| Contacts | Names and phone numbers for clinic staff or user-entered contacts |
| Community | Anonymous community board where participants can communicate with each other on topics of their choosing |
| Resources | Frequently asked questions, links to recovery related information, and scheduling and location information for recovery group meetings |
