# Supplementary material for: Six-month outcomes of the HOPE smartphone application designed to support treatment with medications for opioid use disorder and piloted during an early statewide COVID-19 lockdown
Source: Addict Sci Clin Pract. 2022 Mar 7;17:16. doi: 10.1186/s13722-022-00296-4 (PMC8899792; doi:10.1186/s13722-022-00296-4)
Supplement: Supplementary file 2 — Additional file 2. HOPE app activity. Summary of participant activity on platform during 6-month post-enrollment period. [file 13722_2022_296_MOESM2_ESM.docx]

**Additional file 2: HOPE app activity.** Participant activity is summed for each participant and averaged across cohort participants (N=25) for each month following enrollment. Daily check-in response rates are calculated per participant per month by subtype and averaged for all cohort participants. Active user is defined as a participant using a respective feature one or more times in a given month.

|  | **Month 1** | **Month 2** | **Month 3** | **Month 4** | **Month 5** | **Month 6** |
| --- | --- | --- | --- | --- | --- | --- |
| **Direct provider messaging** | | | | | | |
| Messages sent per user, mean (SD, range) | **7.72**  **(7.04, 0-31)** | **6.16 (6.79, 0-27)** | **4.2 (4.58, 0-15)** | **6.96 (5.53, 0-18)** | **4.88 (5.75, 0-19)** | **1.6 (1.85, 0-5)** |
| Total messages sent by cohort, N | **193** | **154** | **105** | **174** | **122** | **40** |
| N, active users (% cohort) | **22 (88%)** | **21 (84%)** | **18 (72%)** | **21 (84%)** | **16 (64%)** | **13 (52%)** |
| **Community board** | | | | | | |
| CMB posts per user, mean (SD, range) | **0.84 (1.6, 0-7)** | **0.32 (0.9, 0-4)** | **0.32 (0.9, 0-4)** | **0.2 (0.5, 0-2)** | **0.12 (0.4, 0-2)** | **0.04 (0.2, 0-1)** |
| Total posts by cohort, N | **21** | **8** | **8** | **5** | **3** | **1** |
| N, active users (% cohort) | **9 (36%)** | **4 (16%)** | **4 (16%)** | **4 (16%)** | **2 (8%)** | **1 (4%)** |
| **Daily check-ins** | | | | | | |
| Mood, mean % response rate | **88%** | **73%** | **64%** | **59%** | **58%** | **44%** |
| Stress, mean % response rate | **88%** | **73%** | **64%** | **59%** | **57%** | **44%** |
| Medications, mean % response rate | **84%** | **68%** | **61%** | **54%** | **52%** | **40%** |
| Substance use, mean % response rate | **84%** | **68%** | **61%** | **55%** | **52%** | **40%** |
| N, active users (% cohort) | **25 (100%)** | **25 (100%)** | **23 (92%)** | **23 (92%)** | **24 (96%)** | **19 (76%)** |
| **Experiences** | | | | | | |
| Trigger entered, N | **12** | **3** | **1** | **1** | **1** | **0** |
| Encouragement entered, N | **15** | **3** | **0** | **1** | **0** | **0** |
| N, active users (% cohort) | **13 (52%)** | **2 (8%)** | **1 (4%)** | **2 (8%)** | **1 (4%)** | **0 (0%)** |
| **Goals** | | | | | | |
| Working On It, N | **11** | **0** | **0** | **0** | **3** | **0** |
| Achieved, N | **1** | **0** | **0** | **0** | **2** | **0** |
| N, active users (% cohort) | **9 (36%)** | **0 (0%)** | **0 (0%)** | **0 (0%)** | **2 (8%)** | **0 (0%)** |
